# Antibiotics Use in Food Animal Production: Escalation of Antimicrobial Resistance: Where Are We Now in Combating AMR?

**DOI:** 10.3390/medsci9010014

**Published:** 2021-02-21

**Authors:** Vanmathy Kasimanickam, Maadhanki Kasimanickam, Ramanathan Kasimanickam

**Affiliations:** 1 Veterinary Clinical Sciences Department, College of Veterinary Medicine, Washington State University, Pullman, WA 99164, USA; ramkasi@wsu.edu; 2 AARVEE Animal Biotech, LLC, Corvallis, OR 97333, USA; 3 School of Biological and Population Health Sciences College of Public Health & Human Sciences, Oregon State University, Corvallis, OR 97331, USA; kasimanm@oregonstate.edu

**Keywords:** antibiotics, resistance, veterinary medicine, food animal, public health

## Abstract

The use of antibiotics has been very beneficial to human health, animal wellbeing, and food production, however, there are no alternatives to antimicrobials in treating infectious diseases. Their use can contribute to the development of antimicrobial resistance, but the world has realized the need to combat antimicrobial resistance in recent decades due to the continued escalation of the problem jeopardizing human and veterinary medicine and food and environmental safety. Understanding the AMR and judicious use of antimicrobials are critical, and one health approach involving several sectors and multiple disciplines is important to tackle the problem. National, regional, and global action plans have been instigated to tackle the escalation of AMR. Antimicrobials are frequently used in food animal production. Therefore, food animal producers are important participants to prevent overuse and misuse of antimicrobials. Recent regulations to address the challenges have not been perceived well in animal farming communities. More awareness regarding these action plans and understanding the impact of AMR are needed. A nationwide survey of perceptions of food animal producers regarding AMR mitigation approaches should be conducted to evaluate the effectiveness of the current policies regarding antibiotics use and AMR. These outcomes should be incorporated in future policies and awareness campaigns targeting food animal producers.

## 1. Preamble

Raising healthy and safe animals for food production is a high priority for farmers, ranchers, and veterinarians. Despite providing good nutrition, keeping a clean and dry environment with good ventilation, animals being vaccinated to prevent important diseases, and implementing possible biosecurity protocols in farms, trade shows, and marketplaces, animals become sick just like humans. Treatment of sick animals should not be avoided or delayed, and it is everyone’s responsibility to prevent animal sufferings. Antibiotics are as essential in treating bacterial diseases in animals as they are in people. There are no alternatives to antibiotics in treating severe bacterial infections in humans and animals. Antibiotics kill harmful bacteria or limit their growth to stop illnesses from spreading and protect the rest of a herd or flock. Consequently, antibiotics promote good animal health and welfare, contribute to food safety, prevent disease outbreaks, and save lives as well as the livelihoods of farmers. Moreover, antibiotics have been used for non-medicinal purposes as feed efficiency enhancers and growth promoters in agriculturally important animals [1,2]. Since the discovery, antibiotics have not only been used to treat and prevent diseases but also have been widely used in the livestock and poultry industries for efficient production of pork, beef, poultry meat, and other animal products. When antibiotics are used at subtherapeutic levels in feeds, antibiotics improve growth rate and efficiency of feed utilization, reduce mortality and morbidity, and improve reproductive performance [3]. Even though the exact mechanism of how antibiotics enhance the growth is unknown, some of the proposed mechanisms by which antibiotics improve growth include inhibition of subclinical pathogenic bacterial infections; reduction of microbial metabolism products that may negatively affect growth; inhibition of microbial growth, thereby increasing nutrients available to the livestock, and increase in uptake and utilization of nutrients uncompetitively through the intestinal wall [4]. Although the antimicrobials have been used in beef cattle, dairy cattle, sheep, and companion animals, swine and poultry industries have been the primary users of antimicrobials [3]. The use of antibiotics as growth promoters and feed enhancers largely resulted in economic gains [5]. The use of antibiotics, along with practices like vaccination, biosecurity, good husbandry, and nutrition, keep animals protected from serious illnesses. Although antibiotics as feed additives provide benefits to agricultural animal health and welfare, the continuous use at the sub-therapeutic level poses significant risks to public health due to the enrichment of resistant microorganisms. Further, antibiotic residues constitute a threat to human health by entering the food supply chain [6,7,8,9] and the environment [10,11,12,13,14,15]. Risks of antibiotic use in animal feeds have been increasingly documented than benefits. Hence, the world has recognized that antimicrobial resistance is one of the biggest public health threats of the present time.

## 2. Impact of Antimicrobial Resistance

Antimicrobial resistance is present in every country, and more than 2.8 million antibiotic-resistant infections occur in the U.S. each year, and more than 35,000 people die as a result of antibiotic-resistant infections [16]. We are all at risk and we face a silent, slow-growing global threat that kills around 700,000 people worldwide each year due to the uncontrolled rise of superbugs resistant to antibiotics. This threat is on the rise and 10 million people will die every year due to antimicrobial-resistant infections by 2050 unless a substantial global response to the problem of AMR is executed [17,18,19,20]. Antibiotic resistance occurs when bacteria are not killed or their growth is not arrested by these drugs that were previously effective. Although the resistance occurs naturally, misuse of antibiotics in humans and animals can accelerate the process. Antibiotics kill bacteria or prevent proliferation and growth of bacteria by inhibiting cell wall synthesis, impeding protein synthesis, inhibiting nucleic acid production, altering cell membrane properties, and hindering metabolites’ function. Bacteria can escape from these antibiotics’ actions by creating new resistance mechanisms through gene mutations. The new resistance mechanisms continue to emerge, spread globally, and threaten the ability to treat common infectious diseases [21,22,23,24].

## 3. Role of Animal Health Communities

**Figure 1:**
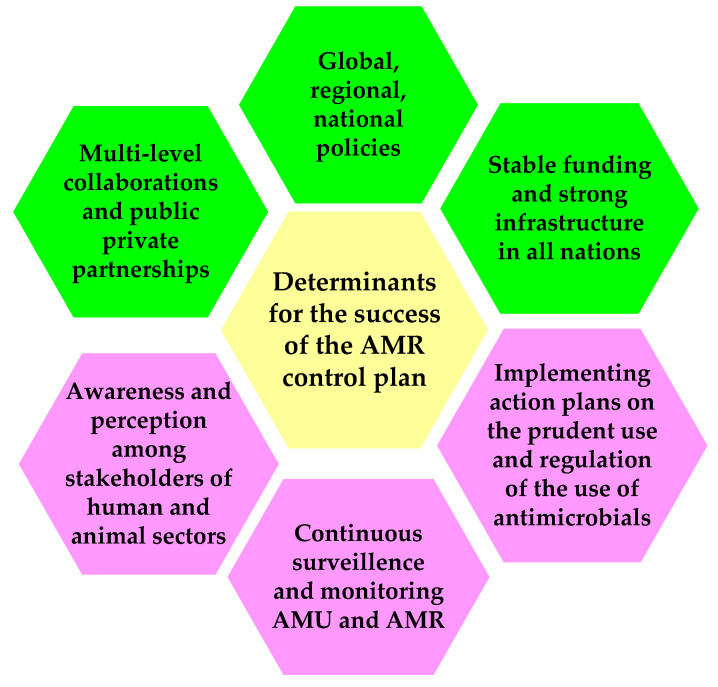
Key determinants deciding the success of the national, regional, and global level coordinated actions to combat AMR by reducing the use of antimicrobials and discovering alternatives.

Although many veterinarians, producers, and animal health stakeholders initially felt that rearing animals “without antibiotics” worsens animal health, welfare, and production efficiency, they are now formally committed to tackling antibiotic resistance and promoting the responsible use of antibiotics [25,26]. Human, animal, and plant health sectors have a collective responsibility to prevent or minimize antimicrobial resistance selection pressures on both human and non-human pathogens. Responsible and judicious use of antibiotics in animals, and thus preserving the effectiveness of antibiotics, are essential. The field of veterinary medicine adopts a unified one health approach to mitigate globally escalating antibiotic resistance, considering human and animal health, and environmental impact equally while protecting and treating animals responsibly and maintaining food safety and security effectively. The magnitude of the antimicrobial resistance threat, and the necessity to curb the escalation of the danger, is widely acknowledged by several governments, international organizations, public health agencies, researchers, and private companies in the same way. Animal health companies and organizations globally pledge to implement five principles and practical actions, protecting animal health and welfare using a unified one health approach, using antibiotics judiciously and sensibly, encouraging disease prevention and increasing access to vaccine products, diagnostic techniques and veterinary expertise, investing in research and development of products for disease prevention and treatment, and increasing knowledge, transparency and communication using science and evidence-based solutions to address these interconnected challenges [25,26,27,28,29,30,31]. Future interventions to optimize antimicrobial use need to focus on the continued emergence of antimicrobial resistance and the evolution of antimicrobial use practices in a changing political and scientific landscape while also contemplating individual motivations and justifications for use. There are several primary determinants (Figure 1) to decide the success of the multi-level coordinated action plans to combat AMR.

## 4. Global Undertakings for Threat of Antimicrobial Resistance

In 2015, the World Health Assembly advocated a global action plan to tackle antimicrobial resistance to ensure the availability of continuous successful treatment and prevention of infectious diseases with quality-assured, effective, and safe medicines to everyone but to use the antimicrobials in a responsible way. The WHO’s five strategic objectives were to improve perception and understanding of antimicrobial resistance; to strengthen knowledge through surveillance and research; to reduce the incidence of infection; to optimize the use of antimicrobial agents; and to develop sustainable investment that fulfills the needs of all countries in capitalizing in new medicines, diagnostic tools, vaccines, and other interventions [32]. The WHO and the European Investment Bank (EIB) have been expanding investment to support the development of antibacterial treatment for public health priorities since 2018. Furthering investment has been discussed in several international conferences, including at the Biocom AMR Conference, the World Health Summit in Berlin, and the World AMR Congress in Washington. In 2019, WHO brought the International Federation of Pharmaceutical Manufacturers and Associations, the EIB, and Wellcome Trust together to launch an initiative, AMR Action Fund [33]. The AMR Action Fund intends to develop 2–4 new antibiotics for patients by 2030 through working with partners to create market conditions that enable sustainable investment in the antibiotic pipeline. The AMR Action Fund initiated by leading pharmaceutical companies and supported by the International Federation of Pharmaceutical Manufacturers and Associations expects to invest more than US$1 billion in smaller biotech companies and provide industry expertise to support the clinical development of novel antibiotics [33].

United Kingdom’s 2019–2024 current national action plan built on its previous 2013–2018 strategy has been formed in consultation with a broad range of stakeholders across various sectors and is affiliated with global plans and frameworks for action to confront antimicrobial resistance within and beyond its own borders. The blueprint has ultimately been designed to ensure progress towards its 20-year vision on antimicrobial resistance by which the escalation of resistance can be effectively contained and controlled. The scheme focuses primarily on reducing the need of, and unintentional exposure to, antimicrobials; optimizing the use of antimicrobials; and investing in research, innovation, supply, and availability [34]. European Union (EU) stands at the forefront to address the regional and global challenges on AMR. In 2016, the EU’s new action plan built on the 2011 action strategy commits to a high level of human health protection. The roadmap on a new EU action plan regarding AMR received contributions from 22 stakeholders and the public [35,36]. Its primary goal is to preserve the effective treatment of infections in humans and animals. The new plan has a framework for sustained, more extensive action to reduce the emergence and spread of AMR, and to enhance the development and availability of novel efficient antimicrobials inside and outside the EU. The main goals of this new plan are based on three main pillars, to assist member states in establishing, implementing, and monitoring their own national One Health action plans on AMR, and to make the EU a best practice region. The framework intends to enhance research, development, and innovation by closing current knowledge gaps, delivering novel solutions and tools to prevent and treat infectious diseases, and improving diagnosis to control the spread of AMR; and to strengthen the EU efforts worldwide to shape the global agenda on AMR and the related risks in an increasingly unified world [37].

The Government of Canada announced a federal framework for action to respond to the threat of AMR in 2014. The coordinated measures were undertaken jointly by the Public Health Agency of Canada (PHAC), Health Canada (HC), the Canadian Food Inspection Agency (CFIA), the Canadian Institutes of Health Research (CIHR), Agriculture and Agri-Food Canada (AAFC), the National Research Council (NRC), and Industry Canada (IC) to prevent, limit, and control the emergence and spread of AMR. Additionally, the Government of Canada has been engaged with international partners to develop and implement a Global Action Plan on AMR [38]. While focusing on surveillance, stewardship, prevention and control of infection, and research and innovation nationally, it is also participating in “transnational network plus 2020” of Joint Programming Initiative on Antimicrobial Resistance (JPIAMR) along with other member countries, France, Italy, Latvia, the Netherlands, Norway, Spain, and Sweden to outline and implement AMR research in the areas of therapeutics, diagnostics, surveillance, transmission, environment, and interventions [39]. The Australian Government has announced Australia’s National Antimicrobial Resistance Strategy: 2020 and Beyond, which was built on their first national AMR Strategy 2015–2019. The 2020 Strategy has an action plan for 20 years, and it continues to coordinate with the WHO’s global action plan on AMR to tackle the challenges of AMR at national and global levels [40].

While developed countries have well-coordinated action plans to reduce AMU and thus prevent AMR, developing and underdeveloped countries are struggling to implement effective intervention strategies since AMU and development of AMR are complicated with several factors, such as inappropriate prescription practices, inadequate client or patient education, unauthorized sale of antimicrobials, improper functioning of drug regulatory mechanisms, and inappropriate use of antimicrobials in food animal production. India’s National Action Plan to tackle longstanding concerns of antibiotic misuse in the human and animal health sectors was released in 2017 by the Union Ministry of Health and Family Welfare [41]. The goals of the action plan were to improve awareness, enhance surveillance measures, focus on prevention and control of infection, design research, develop alternatives to antimicrobials, promote investments, and collaborate activities between various sectors [42,43]. Based on the national plan, various states have begun implementing their State Action Plans in India. However, since the escalation of AMR is made more complex by several factors including lack of awareness and education about AMR, and limited financial allocation for implementation, periodical assessment for effectiveness is necessary, and significant efforts from all stakeholders are needed to achieve these goals. Bangladesh developed a National Action Plan to contain AMR in 2018, however, the country still faces challenges for practical implementation due to poor awareness about AMR, inadequate resources, and absence of national surveillance [44]. AMR preparedness is lacking in sub-Saharan African countries when compared to intervention strategies of developed countries due to a lack of real-time monitoring, surveillance, and regulatory guidelines [45]. Irrational use of antimicrobials is a complex and multifactorial problem in developing countries, and a proper understanding of the problem is necessary for effective implementation strategies to combat AMR.

Veterinary medicinal products, including antimicrobial agents, contribute to a global public good by protecting animal health and welfare, in the knowledge that animal diseases can cause production losses of up to 20%; contributing to food safety, as world population growth increases the demand for high-quality animal protein that found in eggs, meat, and milk; and defending public health, because more than 60% of infectious animal diseases are transmissible to humans. Safeguarding the efficacy of these antimicrobials remains crucial. World Organization for Animal Health (OIE) has been engaged in combating antimicrobial resistance through a one health approach for the past two decades to mitigate antimicrobial resistance to protect human, animal, plant, and environmental health [46]. The OIE approach on antimicrobial resistance and the prudent use of antimicrobials is improving antimicrobial resistance awareness and understanding through targeted communication and advocacy materials, strengthening knowledge on antimicrobial use and the emergence of resistant organisms through research and surveillance, supporting good governance and capacity building on animal health and welfare, stewardship of antimicrobial products, and encouraging the implementation of international standards. The OIE works closely with its member countries, WHO, FAO, and the Codex Alimentarius Commission to develop standards and guidelines to address the risk of the emergence or spread of resistant bacteria that result from the use of antimicrobial agents in food-producing animals. The OIE fourth annual 2019 report on the use of antimicrobial agents intended for use in animals provides the global use of antimicrobial agents adjusted for animal biomass and interprets the overall findings in a global and regional context. With this simultaneous development of quantitative data collection and calculation of animal biomass, this interpretation will allow evaluating global and regional trends on antimicrobial agents intended for use in animals over time. The OIE remains committed to engagement with countries to improve these data, and to refine the methodology for calculating animal biomass to improve guidelines and recommendations for member countries to ensure the responsible and judicious use of antimicrobial agents as good veterinary and animal husbandry practices. Classification of important veterinary antimicrobial agents has been revised in June 2019 by the OIE international committee and classified into three categories, Veterinary Critically Important Antimicrobial Agents (VCIA); Veterinary Highly Important Antimicrobial Agents (VHIA); and Veterinary Important Antimicrobial Agents (VIA) for food-producing animals [47].

Since AMR is a mounting threat to global health and wellbeing globally, region-wide, and nationwide action strategies to combat AMR are being reinvigorated from time to time through synchronized actions of various agencies and different health sectors (Table 1 and Table 2). Although underdeveloped countries struggle to implement these measures due to poor infrastructure and low adaptability by their citizens, economically advanced nations continue to invest in enhancing control measures and searching for alternatives to reduce AMU and AMR.

## 5. US Commitments on Mitigation of Antimicrobial Resistance

US Food and Drug Administration (FDA) has been making efforts to promote the judicious use of medically important antimicrobial drugs in food-producing animals. The FDA released two non-binding documents (guidance for industry; GFI #209 and GFI #213) in 2012 and 2013, respectively, to phase out the use of medically important antimicrobials in food animals for production purposes (to enhance growth or improve feed efficiency), and to implement the therapeutic uses of such drugs (to treat, control, or prevent specific diseases) under the oversight of licensed veterinarians to protect public health. The GFI #209 is about the judicious use of medically important antimicrobial drugs in food-producing animals with collaboration linking the public, and the public health, animal health, and animal agriculture communities. The GFI #213 depicts new animal drugs and new animal drug combination products, administered in or on medicated feed or drinking water of food-producing animals: recommendations for drug sponsors for voluntarily aligning product use conditions with GFI #209. FDA believed this collaborative approach would be most effective and speedy in implementing changes while protecting both public and animal health and making these antimicrobial drugs available for the purposes of treating, controlling, or preventing diseases in food-producing animals. In 2015, the FDA released the Veterinary Feed Directive (VFD) final rule that states how veterinarians authorize the use of VFD-assigned animal drugs in feed. The revised rule enhances the efficiency of the VFD process and facilitates the implementation of GFI #213 to transition the marketing status of antimicrobial drug products from over-the-counter use to VFD use under the direction of a licensed veterinarian. In 2015, the Obama Administration implemented the National Action Plan to combat antibiotic-resistant bacteria and the goals of the action plan are reducing the emergence of resistant bacteria and thwarting the spread of resistant infections, strengthening national One Health surveillance to combat antimicrobial resistance, advancing development and use of state-of-the-art diagnostic tests for identification and characterization of resistant microbes, and hastening basic and applied research and development for new antibiotics, alternative therapeutics, and vaccines. In 2017, the FDA fully implemented the VFD final rule to facilitate the judicious use of medically important antimicrobials in food-producing animals, although perceptions about the VFD among animal producers are inadequate [48,49,50].

FDA has been working in partnership with USDA and CDC in collecting data on antimicrobial resistance among foodborne pathogens as part of the National Antimicrobial Resistance Monitoring System. FDA in collaboration with AVMA, other veterinary associations, and animal producer organizations have been executing outreach activities for animal producers and veterinarians to understand the role that they can play in ensuring the appropriate and judicious use of these important drugs. Once manufacturers voluntarily adopt these adjustments, medically important antimicrobial drugs can no longer be used for growth promotion and feed efficiency enhancement, and their continued use to treat, control, or prevent diseases in food-producing animals will require a prescription from a licensed veterinarian [51].

In 2018, the US Food and Drug Administration’s Center for Veterinary Medicine released a five-year action plan (2019–2023) for supporting antimicrobial stewardship in veterinary settings. This blueprint builds upon the strategies to eliminate production uses of medically important antimicrobials and to adopt all therapeutic uses of these drugs under the oversight of licensed veterinarians. In other words, medically important antimicrobial drugs should only be used in animals when these drugs are needed for the treatment and control. This five-year plan includes activities like supporting antimicrobial stewardship in veterinary settings, human food safety assessment for new antimicrobials, guidance on appropriate and judicious use of antimicrobials, direction on phasing out production uses, revises to the veterinary feed directive, enhancing surveillance activities (NARMS), education and outreach, expanding research activities, partnership with international partners focused on antimicrobial resistance, withdrawal of enrofloxacin for Poultry, and gathering and analyzing better antimicrobial use information [52].

Although USDA is not the lead governing agency, the USDA plays a critical role in addressing challenges associated with the antimicrobial use in agricultural animals and antimicrobial resistance through partnerships with stakeholders and its key agencies, the Agricultural Research Service (ARS), the Animal and Plant Health Inspection Service (APHIS), the Economic Research Service (ERS), the Food Safety and Inspection Service (FSIS), the National Agricultural Statistics Service (NASS), and the National Institute of Food and Agriculture (NIFA). The USDA’s action plan recommends a voluntary thorough, integrated approach to expand future surveillance, research and development, and education, extension, and outreach activities [53]. Its three main objectives are to determine and shape the patterns, purposes, and impacts of antibiotic use in food-producing animals, scrutinize antibiotic drug susceptibilities of bacteria in food-producing animals, production environments, and meat and poultry, and detect possible management practices, alternatives to antibiotic use, and other mitigation strategies to reduce AMR linked to food-producing animals and their production environments. Since the USDA has comprehensive knowledge of the management practices and technologies associated with animal health, welfare, productivity, and food safety, and has been involved in surveillance, basic and applied research, and education and outreach to assess levels of AMR, to build effective mitigation approaches to tackle AMR, and to assist animal producers to implement these approaches, the USDA is uniquely placed to contribute to the scientific knowledge of AMR, in pertinent to the role of antimicrobial use in livestock. The successful implementation of this action plan would provide and disseminate science-based information to the veterinary and animal agricultural communities to implement effective mitigation strategies that will prolong the effectiveness of antibiotics to use in both animals and people.

## 6. Perception of Regulations Regarding the Use of Antimicrobials and Awareness of Antimicrobial Resistance in the Field

Overuse and misuse of antimicrobials contribute to the development of antimicrobial resistance, including those critically important drugs for treating human disease. Antibiotic use in food animals promotes the development of antibiotic-resistant bacteria that can be transmitted to humans through several pathways such as consumption of contaminated animal products [54,55,56,57], eating of crops contaminated by manure fertilizers or water containing animal feces [58,59,60], direct contact with food animals [61,62,63], and contact with the contaminated environment by effluent and manure from food animal farms [64]. It is very critical to optimize antibiotic use in animal agriculture in order to minimize the development of antibiotic resistance considering the various potential routes of human exposure to antibiotic-resistant bacteria from animal agriculture. Concerns have been raised that the push to reduce antibiotic use could result in unintended negative consequences on food safety, animal health, production, and the economic viability of animal farming. Agricultural animal producers are important stakeholders, and when they are likely to experience any negative consequences that arise as a result of attempts to alter antibiotic use, the adverse outcomes should be addressed through discussion [65,66]. Despite the important responsibility of food animal producers to reduce antibiotic use in animal agriculture, little or vague understanding exists regarding antibiotics use and antibiotics resistance, and recent regional, national, and international regulations to address the challenges associated with AMR have not been perceived well. As an instance, many food animal producers are either not aware or slightly aware of the VFD statement in the US or other similar national rules in other countries, and some of the farmers are perceived these regulations as not useful [49,67,68,69,70,71,72,73,74]. More awareness regarding the VFD or other similar plans in other countries and understanding the impact of AMR are needed among agricultural animal producers. A nationwide survey of the perceptions of food animal producers regarding the VFD or other similar rule should be conducted via personal interviews, questionnaires, and direct observations to evaluate the effectiveness of the current policies and strategies regarding antibiotics use and AMR, and these outcomes should be incorporated in future policymaking and implementation, and educational initiatives and awareness campaigns targeting food animal producers [49,75,76,77,78].

## 7. Conclusions

Since its discovery in the 1930s, antibiotics have been saving human and animal lives from life-threatening infections. There were no alternatives to antimicrobials in the treatment of infectious diseases. Additionally, the vast majority of antibiotics have been used in food animal production for promoting weight gain and enhancing feed efficiency. But the use of antimicrobials contributes to antimicrobial resistance that jeopardizes human and animal health and food and environmental safety. Although antibiotics resistance had been recognized in the 1940s, the world realized the continued escalation of the AMR threat in recent decades. Overuse and misuse of antimicrobials should be halted, and new alternatives to antimicrobials should be searched to mitigate the AMR. National, regional, and global levels of regulations have been instigated in recent years. Although this action plans largely vary among countries and regions, each plan has one main goal to achieve to combat AMR via implementing judicious use of antimicrobials, increasing hygienic and biosecurity protocols to prevent diseases, and developing new alternatives to antibiotics such as vaccines, probiotics, and feed additives. The effectiveness of these policies and rules is still in question. Since antimicrobials are frequently used in food animal production, food animal producers are important participants to prevent overuse and misuse of antimicrobials. Recent regulations to address the challenges of AMR have not been perceived well among agricultural animal farming communities. More awareness regarding these action plans and understanding the impact of AMR are needed. Based on the current perception level, additional measures should be incorporated in future policies and awareness campaigns targeting food animal producers.

## Figures and Tables

**Table 1:** Predominant global and regional action strategies to combat AMR at global and regional levels in addition to the WHO universal action plan (https://www.who.int/antimicrobial-resistance/global-action-plan/ongoing_activities/en/, accessed on 21 February 2021).

Strategies	Countries Involved	Year of Establishment of Action Plan and Website
Global Antibiotic Resistance Partnership (GARP)	India, Kenya, South Africa, Vietnam, Mozambique, Nepal, Tanzania, and Uganda	2009 https://cddep.org/projects/global-antibiotic-resistance-partnership/ (accessed on 21 February 2021)
Global Health Security Agenda (GHSA)	Canada, the European Union (EU), Norway, and the United States (US)	2014 https://www.hhs.gov/about/agencies/oga/global-health-security/antimicrobial-resistance/index.html (accessed on 21 February 2021)
Jaipur Declaration on AMR	India and other countries in Southeast Asia	2011 https://www.who.int/southeastasia/health-topics/antimicrobial-resistance (accessed on 21 February 2021)
Strategic action plan to control AMR in the Asia-Pacific region	Countries in the Asia Pacific region	2010 https://www.apec.org/Publications/2011/10/International-initiatives-to-control-antimicrobial-resistance-in-the-Asia-Pacific-region (accessed on 21 February 2021)
European Commission Action Plan against the rising threats from AMR	Countries in the EC	2011 https://ec.europa.eu/health/antimicrobial-resistance/eu-action-on-antimicrobial-resistance_en (accessed on 21 February 2021)
Transatlantic Task Force on Antimicrobial resistance (TATFAR)	Canada, the European Union (EU), Norway, and the United States (US)	2009 https://www.cdc.gov/drugresistance/tatfar/index.html (accessed on 21 February 2021)

**Table 2:** National action plans of various countries to combat AMR through coordinated measures from different sectors. Note that only freely available information is presented to represent the action plans to combat AMR (https://www.who.int/antimicrobial-resistance/global-action-plan/ongoing_activities/en/, accessed on 21 February 2021).

Countries	Objectives of AMR Control Strategies	Year of Launching of Action Plan to Combat AMR and Website
Australia	Minimizing development and spread of AMR; Assurance of availability of effective antimicrobials.	2015–2019—first national plan; 2020 and beyond—subsequent national plan. www.amr.gov.au/australias-response/national-amr-strategy (accessed on 21 February 2021)
Canada	Reacting to the threat of AMR to protect people, animals, and the environment in Canada.	2014. https://www.canada.ca/en/public-health/services/antibiotic-antimicrobial-resistance/antimicrobial-resistance-use-canada-federal-framework-action.html (accessed on 21 February 2021)
Ethiopia	Effective prevention and containment of AMR in humans, animals, agriculture, and the environment in Ethiopia.	2009–2014—initial plan; 2015–2020—second refreshment. http://www.fmhaca.gov.et/wp-content/uploads/2019/03/Strategy-for-the-Prevention-and-Containment-of-AMR-in-Ethiopia-Oct-2015.pdf (accessed on 21 February 2021)
France	Tackling AMR in human medicine and veterinary medicine promoting good practices and awareness among all stakeholders.	2000—three refreshments in human medicine and one plan in veterinary medicine since 2000. http://resistancecontrol.info/2016/government-engagement/the-french-approach-to-fighting-antibiotic-resistance-a-constant-and-coordinated-effort-since-2000/ (accessed on 21 February 2021)
Germany	Prevention of occurrence and spread of AMR adopting measures in human and veterinary medicine.	2008 initiative; DART2020 continuation strategy. https://www.bmel.de/EN/topics/animals/animal-health/DART2020.html (accessed on 21 February 2021)
India	Construction of implementable recommendations to tackle AMR involving human, animal, and environmental sectors in India	2012 initiative with a five-year plan. http://chennaideclaration.org/ (accessed on 21 February 2021)
Ireland	Implementing policies and actions to prevent, monitor and tackle AMR across the health, animal, and environmental sectors	2009 initiative; 2017–2020 subsequent national plan. https://www.hse.ie/eng/services/list/2/gp/antibiotic-prescribing/ (accessed on 21 February 2021)
Norway	Adopting appropriate use of antibiotics across the sectors, human, animal, aquatic and environmental sectors.	2008–2012 initial plan; 2015–2020 subsequent strategy. https://www.regjeringen.no/en/search/id86008/?term=antimicrobial+resistance (accessed on 21 February 2021)
Sweden	Raising general awareness on AMR, enhancing hygiene in the community and health care facilities, and using antibiotics rationally rational use of antibiotics.	2000 plan was based on Strama 1995. Overall use in Sweden has been significantly reduced. https://www.government.se/contentassets/168838e186de455ca7fe868bee92d209/swedish-strategy-to-combat-antibiotic-resistance.pdf (accessed on 21 February 2021)
Switzerland	Safeguarding long-term efficacy of antibiotics for human and animal diseases consulting all interested stakeholders.	2015. http://resistancecontrol.info/2018-frontpage/2018-2/the-swiss-recipe-for-containing-antimicrobial-resistance/ (accessed on 21 February 2021)
United Kingdom	Advancing towards the 20-year vision on AMR focusing on reducing need of antimicrobials, optimizing use of antimicrobials, and investing in innovation, supply, and access.	2013–2018 initial strategy; 2019–2024 subsequent plan. https://www.gov.uk/government/publications/uk-5-year-action-plan-for-antimicrobial-resistance-2019-to-2024 (accessed on 21 February 2021)
United States	Highlighting prevention and control of infections to slow the spread of AMR through multi-sectors coordinated approach.	2013—release of AMR threat report by CDC; 2014—establishment of federal task force; 2015—first national action plan; 2020—second national strategy. https://aspe.hhs.gov/pdf-report/carb-plan-2020-2025 (accessed on 21 February 2021)

## Data Availability

No new data were created or analyzed in this review. Therefore, the data sharing is not applicable to this review article.
